# Neratinib overcomes trastuzumab resistance in HER2 amplified breast cancer

**DOI:** 10.18632/oncotarget.1148

**Published:** 2013-07-25

**Authors:** Alexandra Canonici, Merel Gijsen, Maeve Mullooly, Ruth Bennett, Noujoude Bouguern, Kasper Pedersen, Neil A O'Brien, Ioannis Roxanis, Ji-Liang Li, Esther Bridge, Richard Finn, Dennis Slamon, Patricia McGowan, Michael J. Duffy, Norma O'Donovan, John Crown, Anthony Kong

**Affiliations:** ^1^ National Institute for Cellular Biotechnology, Dublin City University, Dublin, Ireland; ^2^ Human Epidermal Growth Factor Group, University of Oxford, UK; ^3^ Growth Factor Group, Department of Oncology, Weatherall Institute of Molecular Medicine, University of Oxford, UK; ^4^ Clinical Research Centre, St. Vincent's University Hospital, and UCD School of Medicine and Medical Science, Conway Institute, University College Dublin, Dublin, Ireland; ^5^ Division of Hematology-Oncology, Department of Medicine, David Geffen School of Medicine, University of California at Los Angeles, Los Angeles, California; ^6^ Department of Cellular Pathology, Oxford University Hospitals and Oxford Biomedical Research Centre, Oxford, United Kingdom

**Keywords:** breast cancer, HER2/ErbB2, trastuzumab (Herceptin), neratinib, panHER inhibitor

## Abstract

Trastuzumab has been shown to improve the survival outcomes of HER2 positive breast cancer patients. However, a significant proportion of HER2-positive patients are either inherently resistant or develop resistance to trastuzumab. We assessed the effects of neratinib, an irreversible panHER inhibitor, in a panel of 36 breast cancer cell lines. We further assessed its effects with or without trastuzumab in several sensitive and resistant breast cancer cells as well as a BT474 xenograft model. We confirmed that neratinib was significantly more active in HER2-amplified than HER2 non-amplified cell lines. Neratinib decreased the activation of the 4 HER receptors and inhibited downstream pathways. However, HER3 and Akt were reactivated at 24 hours, which was prevented by the combination of trastuzumab and neratinib. Neratinib also decreased pHER2 and pHER3 in acquired trastuzumab resistant cells. Neratinib in combination with trastuzumab had a greater growth inhibitory effect than either drug alone in 4 HER2 positive cell lines. Furthermore, trastuzumab in combination with neratinib was growth inhibitory in SKBR3 and BT474 cells which had acquired resistance to trastuzumab as well as in a BT474 xenograft model. Innately trastuzumab resistant cell lines showed sensitivity to neratinib, but the combination did not enhance response compared to neratinib alone. Levels of HER2 and phospho-HER2 showed a direct correlation with sensitivity to neratinib. Our data indicate that neratinib is an effective anti-HER2 therapy and counteracted both innate and acquired trastuzumab resistance in HER2 positive breast cancer. Our results suggest that combined treatment with trastuzumab and neratinib is likely to be more effective than either treatment alone for both trastuzumab-sensitive breast cancer as well as HER2-positive tumors with acquired resistance to trastuzumab.

## INTRODUCTION

HER2 is a member of the human epidermal growth factor receptor (HER) family which also includes EGFR (HER1), HER3 and HER4 [1]. The HER proteins are receptor tyrosine kinases which consist of an extracellular domain, an α-helical transmembrane region and an intracellular tyrosine kinase domain [2]. The crystal structure of HER2 shows that the extracellular domain is constitutively in an ‘open’ conformation, ready for dimerization [3]. Although HER2 has no known ligand, it is the preferred heterodimerization partner of the other HER receptors and is involved in the lateral transmission of signals between other HER family receptors [4]. The HER2/HER3 dimer is considered to be the most potent with regards to strength of interaction, ligand-induced tyrosine phosphorylation and downstream signaling [5, 6]. HER2 signaling promotes cell proliferation via the RAS-ERK pathway and inhibits cell death via the PI3K-Akt-mTOR pathway [7]. Over-expression of HER2 occurs in approximately 20% of breast cancers and is associated with a poor prognosis [8, 9].

Trastuzumab is a humanized monoclonal antibody which binds to extracellular domain IV of HER2. It has been shown to increase survival in HER2 positive metastatic and early breast cancer patients, especially when given in combination with chemotherapy [10, 11]. Trastuzumab is thought to exert its anti-tumor activity partly by accelerating the internalization and degradation of HER2 and thus blocking downstream signaling. In addition, it may also act by mediating antibody-dependent-cellular cytotoxicity [12]. However, many patients do not respond to trastuzumab and the majority who initially respond, progress within a year of initiating therapy [13]. Understanding the mechanisms responsible for resistance to trastuzumab is therefore crucial for the development of new therapeutic strategies and improving patient outcome.

Based on preclinical studies, mechanisms of primary and acquired resistance to trastuzumab include (i) activation of PI3K/Akt signaling due to PTEN loss or PI3KCA mutations [14, 15], (ii) the presence of p95HER2, a truncated form of HER2 [16]; (iii) interaction of HER2 with other receptors such as IGF-IR and c-Met [17, 18] and (iv) activation of HER receptors by an Akt negative feedback loop [19]. Other proposed mechanisms include the constitutive activation of the autophagy-related gene 12 (ATG12) [20], the presence of trastuzumab-refractory breast cancer stem cells (CSCs) [21], and the steric hindrance caused by association of HER2 with other cell surface signaling proteins, i.e. integrins β1, CD44, MUC1 and MUC4 [22].

In an attempt to circumvent resistance to trastuzumab, anti-HER2 tyrosine kinase inhibitors (TKIs) were introduced. The first TKI to show activity in HER2 positive breast cancer patients was the dual EGFR/HER2 reversible TKI, lapatinib [23]. In patients who were resistant to trastuzumab, the combination of lapatinib with capecitabine increased median time to progression compared to capecitabine alone [23]. As a result, lapatinib with capecitabine is currently licensed for use in patients who have previously failed trastuzumab. Clinical trials have shown that the addition of trastuzumab and lapatinib to chemotherapy achieved a higher pathological complete response than either trastuzumab or lapatinib alone [24, 25].

To further enhance HER2 inhibition, irreversible TKIs, such as neratinib, afatinib and dacomitinib have been developed. Neratinib, is an irreversible inhibitor of EGFR, HER2 and HER4 tyrosine kinase activity [26] shown to have promising preclinical activity against HER2-overexpressing cell lines [27]. Indeed, it is currently undergoing several phase 3 clinical trials [28, 29]. Since we have previously shown that trastuzumab-induced activation of all HER receptors may contribute to trastuzumab resistance [19], we assessed the effects of neratinib, to determine if it can improve response and overcome resistance to trastuzumab, in sensitive and resistant HER2 amplified breast cancer cells.

## RESULTS

### Neratinib sensitivity in a panel of breast cancer cell lines

**Figure 1:**
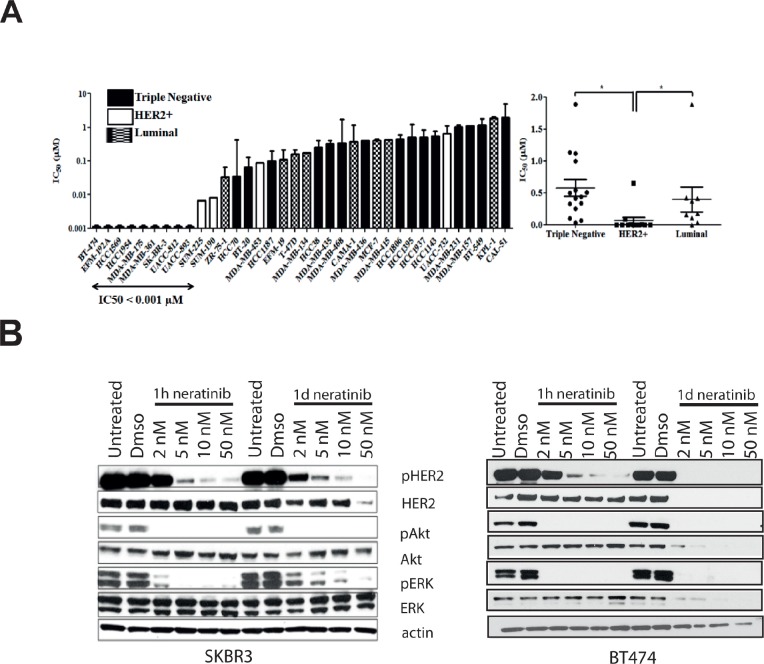
Neratinib is most effective in HER2 positive breast cell lines and reduces HER2 phosphorylation A) Neratinib IC_50_ values (μM) and breast cancer cell type. Cell lines are color coded by subtype: white bars - HER2 amplified; black bars – triple negative; stripes – luminal. Differences between the cell line subtypes were analysed by Mann-Whitney U test. * p <0.05. B) BT474 (right) and SKBR3 (left) cell were treated for 1 hour or 1 day with increasing concentrations of neratinib. After lysis, protein levels were assessed using western blotting techniques.

We determined sensitivity to neratinib in a panel of 36 breast cancer cell lines, including HER2 positive, luminal and basal-like cell lines (Figure 1A left panel, Supplementary Table 1). The HER2 positive cell lines (n=12) were significantly more sensitive to neratinib, than either the triple negative (n=15, p=0.0002) or luminal cell lines (n=9, p=0.0055) (Figure 1A, right panel). One luminal cell line however, MDA-MB-175, showed sensitivity to neratinib (IC_50_ value < 0.001 μM), while one of the HER2 positive cell lines, UACC-732, was relatively insensitive to neratinib (IC_50_ value = 0.65 μM). The IC_50_ values for neratinib were lower than those previously published for lapatinib in the panel of HER2 positive cell lines [30]. The results were also confirmed by direct comparison of the two drugs in SKBR3 and BT474 cells, and both cell lines showed greater sensitivity to neratinib than lapatinib (data not shown).

### Neratinib decreases pHER2 in a dose-dependent manner in HER2 amplified breast cancer cells

As the HER2 positive cell lines were significantly more sensitive to neratinib, we investigated the effect of neratinib on HER2 signalling in two HER2 positive breast cell lines. One hour of neratinib (2 nM) treatment did not decrease pHER2 levels despite inhibition of pAkt and pERK (Figure 1B). However, pHER2 was inhibited after 24 hours (Figure 1B). A dose dependent inhibition of pHER2 was observed with increasing concentrations of neratinib in both cell lines (Figure 1B). Although total HER2 levels were not altered by treatment with neratinib for 1 hour, by 24 hours HER2 level was reduced in response to 50 nM neratinib in SKBR3 and was undetectable in BT474 cells.

Reactivation of pERK was observed after 24 hours of neratinib treatment in SKBR3 cells, although the highest concentration of neratinib (50 nM) inhibited this reactivation (Figure 1B). The reactivation of pERK was not seen in BT474 cells, which are more sensitive than SKBR3 cells (Figure 1B). The results suggest that neratinib effectively inhibits HER2 activation and downstream signaling in HER2 positive breast cell lines.

### Combined treatment with neratinib and trastuzumab has an additive effect

**Figure 2:**
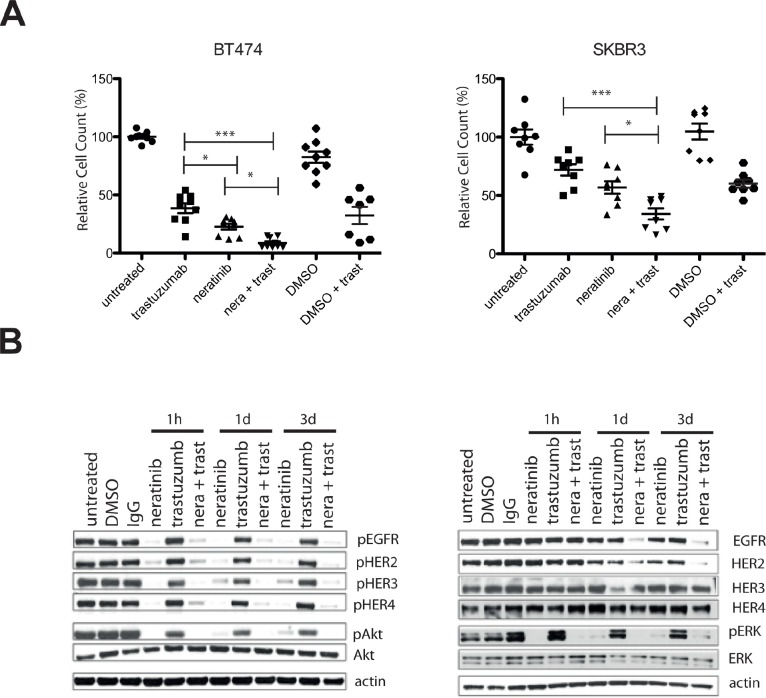
Combination of neratinib and trastuzumab has an additive effect and prevents re-activation of pHER3 and pAkt A) BT474 (left) and SKBR3 (right) cells are treated for 5 days with either 40 μg/ml trastuzumab or 2 nM neratinib or their combination (n=3, both technical and biological replicates are represented in the figures). DMSO is used as a vehicle control for neratinib. Cells were trypsinized and counted using a cell counter. The differences in means of the cell count between the groups were analysed by Anova with Bonferroni's multiple comparison test and statistically significant changes were represented by asterisks (*, P ≤ 0.05; ***, P ≤ 0.001). B) SKBR3 cells were treated with 2 nM neratinb, 40 μg/ml trastuzumab or their combination for the indicated times before the cells were lysed and analysed by western blot.

To assess the effect of trastuzumab with neratinib, SKBR3 and BT474 cells were treated with 40 μg/ml trastuzumab, 2 nM neratinib or the combination for six days. The combination treatment was significantly more effective in reducing cell numbers compared to trastuzumab alone (p< 0.001) or neratinib alone (p< 0.05) in both cells lines (Figure 2A). To understand the enhanced response to the combination, a time-course experiment was performed to assess the effect of the combination on HER receptor activation and downstream signaling. Figure 2B shows the results after three days of treatment in the SKBR3 cells (data for BT474 cells are not shown as there were insufficient cells left after three days of the combination treatment). Trastuzumab decreased pHER3 and pAkt only minimally after 1 hour and did not decrease pEGFR, pHER2, pHER4 or pERK at the timepoints tested (Figure 2B and Supplementary Figure 1). In contrast, 1-hour neratinib treatment inhibited phosphorylation of EGFR, HER2, HER3 and HER4 as well as pAkt and pERK levels (Figure 2B and Supplementary Figure 1). However, reactivation of HER3 and Akt phosphorylation was observed after 24 hours (Figure 2B and Supplementary Figure 1). The combination of trastuzumab and neratinib prevented reactivation of pHER3 and pAkt and downregulated EGFR and HER2 at 3 days (Figure 2B and Supplementary Figure 1).

### Neratinib is effective in acquired trastuzumab resistant HER2 amplified cells

**Figure 3:**
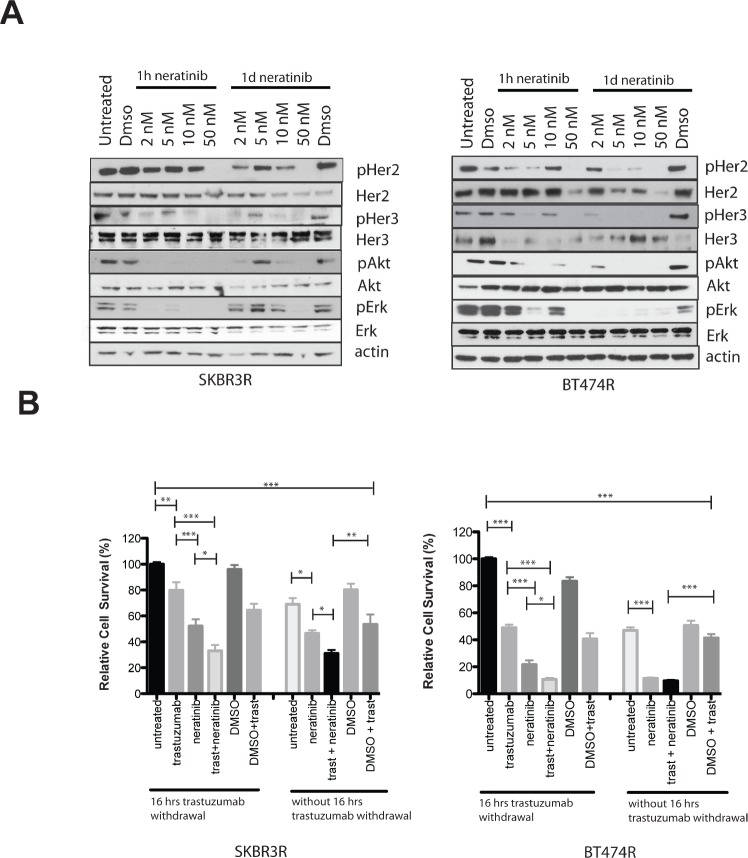
Trastuzumab resistant cells are sensitive to neratinib treatment A) Trastuzumab resistant SKBR3 (SKBR3R, left) or BT474R (right) cells were treated for 1 hour or 1 day with increasing doses of neratinib. The cells were continuously kept in media with 40 μg/ml trastuzumab. Cells were lysed and protein levels analysed by western blot. B) SKBR3R (left) and BT474R (right) cells were treated for 5 days with 2 nM of neratinib, 40ug/ml trastuzumab or their combination. Cells were trypsinized and counted using a cell counter. Cells were either seeded out with 40 μg/ml trastuzumab in the media or plated out overnight without trastuzumab for 16 hours in the media before treatment was commended the next day. The differences in means of the cell count between the groups were analysed by Anova with Bonferroni's multiple comparison test and statistically significant changes were represented by asterisks (*, P ≤ 0.05; **, P ≤ 0.01; ***, P ≤ 0.001).

We tested the effect of neratinib on acquired trastuzumab resistant BT474 (BT474R) and SKBR3 (SKBR3R) cells. With increasing doses, neratinib inhibited pHER2 and pHER3 as well as downstream pathways in both cell lines at 1 hour and 1 day (Figure 3A). We also assessed the effect of trastuzumab withdrawal and/or neratinib treatment in resistant cells. In the BT474R and SKBR3R cells, withdrawal of trastuzumab increased the cell count significantly, compared to trastuzumab replacement or continuous treatment with trastuzumab (Figure 3B). Neratinib monotherapy decreased cell numbers to a greater extent than trastuzumab replacement, in both resistant cell lines (Figure 3B). Furthermore, the combination of trastuzumab and neratinib had a greater growth inhibitory effect than either drug alone, with or without trastuzumab withdrawal in SKBR3R (Figure 3B).

### Combined treatment with neratinib and trastuzumab in a panel of HER2 amplified cell lines

**Figure 4:**
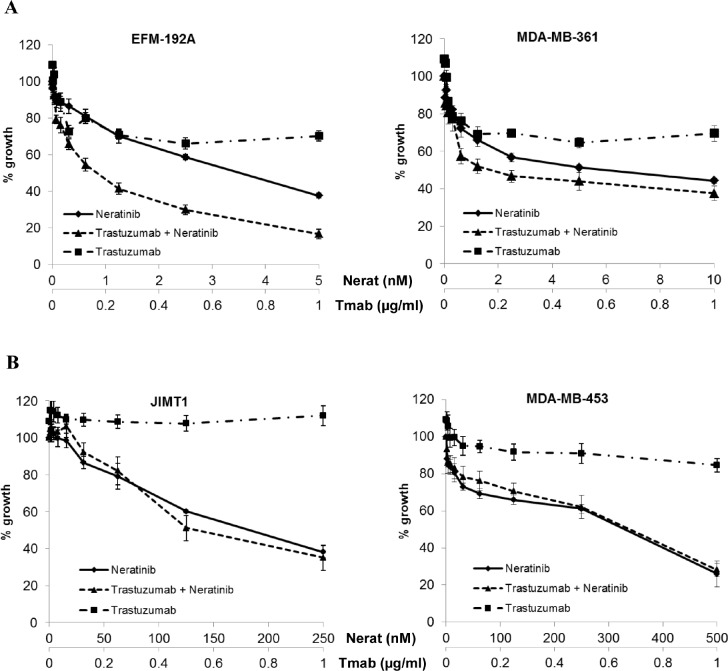
Combined neratinib and trastuzumab treatment in cell line models with varying trastuzumab sensitivity and resistance Sensitivity to trastuzumab and neratinib in A) two trastuzumab moderately sensitive and B) two innately trastuzumab resistant HER2 positive breast cancer cell lines. Cells were treated with neratinib alone, trastuzumab alone or the combination at a fixed ratio for 5 days.

Based on the additive interaction between neratinib and trastuzumab observed in the SKBR3 and BT474 cell lines, we extended the analysis of the combination treatment to 7 additional HER2 amplified breast cancer cell lines, including cell line models of innate trastuzumab resistance. In the MDA-MB-361 and EFM-192-A cell lines, which are moderately sensitive to trastuzumab, the combination of neratinib and trastuzumab showed greater inhibition of growth than either of the drugs alone (Figure 4A). However in the trastuzumab-resistant cell lines (JIMT1, MDA-MB-453, HCC1419, HCC1954, UACC732) the combination treatment showed no enhancement compared to neratinib alone (Figure 4B and Supplementary Figure 2).

### Response to neratinib correlates with HER2 and pHER2 in HER2 amplified breast cancer cell lines

**Figure 5:**
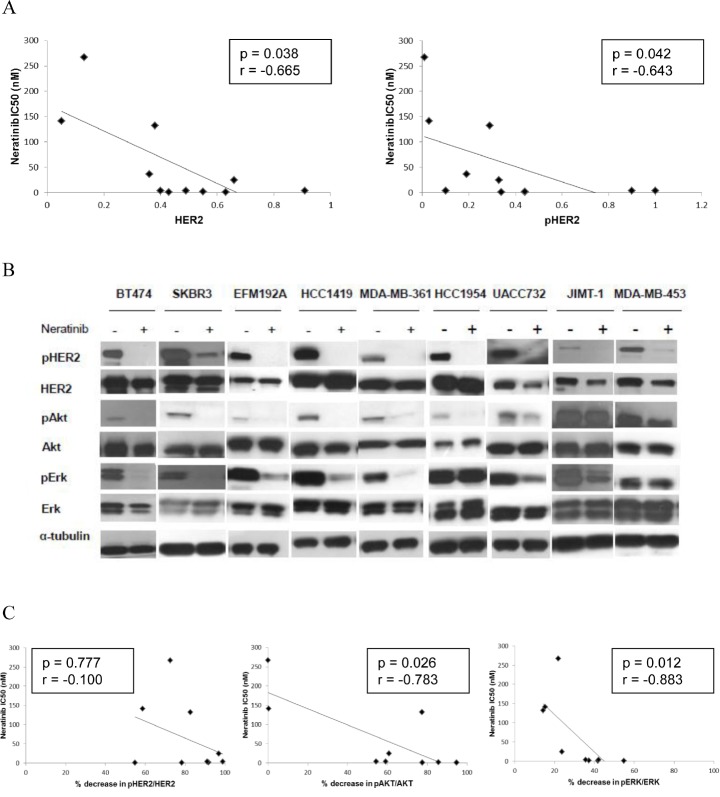
Levels of HER2 and pHER2 predict response to neratinib A) Scatter plots showing the relationship between the levels of both HER2 protein and pHER2 (determined by western blotting) and neratinib sensitivity (IC_50_). B) The effects of neratinib on expression and phosphorylation of HER2, Akt and ERK in HER2 positive breast cancer cell lines. C) Scatter plots showing the relationship between the neratinib (200 nM) induced decrease in phosphorylation of HER2, Akt and ERK and neratinib sensitivity (IC_50_) in 9 HER2 positive breast cancer cell lines.

In order to identify potential predictive biomarkers for neratinib sensitivity in HER2 positive breast cancer, we correlated the neratinib IC_50_ values with a panel of potential biomarkers previously measured by O'Brien et al [30] to determine their relationship with response/resistance to lapatinib and/or trastuzumab. In the panel of 11 HER2 positive breast cancer cell lines, higher baseline levels of both HER2 and phosphorylated HER2 significantly correlated with response to neratinib (p=0.038 and 0.042 respectively) (Figure 5A). Neratinib sensitivity was not significantly associated with p95-HER2, EGFR, HER3, Akt, ERK, PTEN, PI3K mutation status or ER status (Supplementary Table 2).

### Correlation between the effect of neratinib on cell signaling and biomarkers

In order to examine potential pharmacodynamic biomarkers of response to neratinib, we tested the effects of neratinib (200 nM) on HER2 and downstream signalling in a panel of HER2 amplified cell lines, with varying sensitivity to neratinib (Figure 5B). In all cell lines tested, neratinib significantly reduced phosphorylation of HER2, although the level of reduction did not correlate with sensitivity to neratinib (Figure 5C). Neratinib inhibited phosphorylation of Akt and ERK to a greater extent in cell lines which are more sensitive to neratinib (pAkt: p=0.026; pERK: p=0.012).

### Combined treatment with trastuzumab and neratinib is additive in a BT474 xenograft model

**Figure 6:**
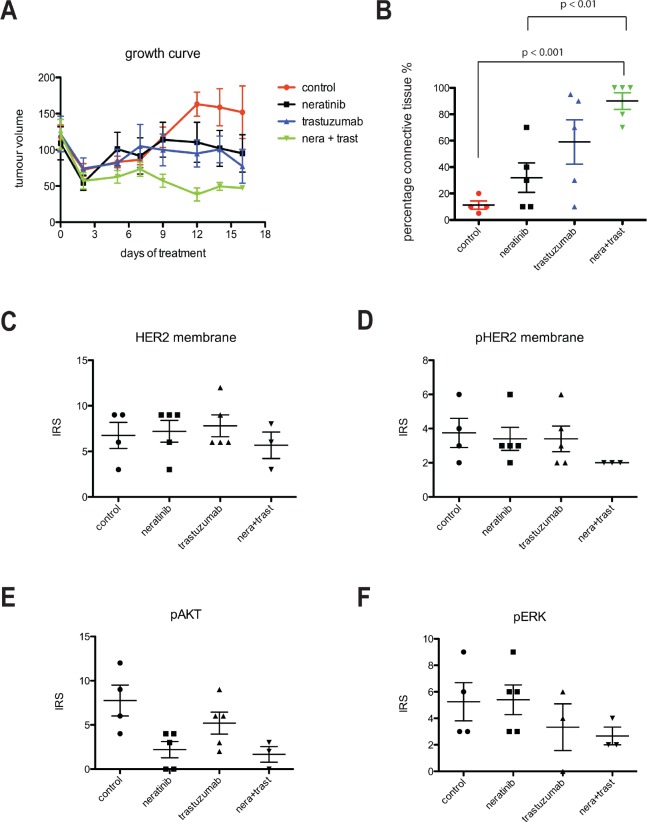
Combination of trastuzumab and neratinib was additive in tumor growth inhibition in BT474 xenograft model A) Left, Mice bearing BT474 xenograft tumors were treated with either control (vehicle), neratinib, trastuzumab or their combination for 16 days. During this time, tumor size was measured in each mouse and the tumor volume calculated. B) At the end of the experiment from A, tumor samples were collected and embedded in paraffin. The slides were cut and analysed for the percentage of connective tissue per section. The differences in means of the percentage of connective tissue between the groups were analyzed by Anova with Bonferroni's multiple comparison test and statistically significant changes were indicated in the figure. C-F), Sections were cut from paraffin-embedded xenograft samples and stained for HER2, phosphorylated HER2 (pHER2), phosphorylated Akt (pAkt) and phosphorylated ERK (pERK). Sections were then scored for the intensity and percentage of staining. The IRS scoring from each condition was shown in the figures.

We further examined the effect of neratinib and/or trastuzumab in a BT474 xenograft model and showed that the combination treatment resulted in the greatest tumour inhibition (Figure 6A), correlated with the final tumor volume *in vivo* among the four groups (vehicle control vs combination group, p< 0.05; all other comparisons, p > 0.05) (Supplementary Figure 3A left panel). The combination treatment also resulted in the smallest tumor weight *ex-vivo* (vehicle control vs combination group, p< 0.05; all other comparisons, p > 0.05) (Supplementary Figure 3A right panel) and with higher percentage of connective tissue *ex-vivo* compared to vehicle control (p< 0.001) or neratinib alone (p< 0.01) (Figure 6B).

Immunohistochemical (IHC) staining in the xenograft tumors showed no statistically difference in the levels of membrane HER2 and pHER2 between any of the groups although the trastuzumab and neratinib combination treatment showed the lowest IRS scoring for pHER2 staining (Figure 6C and 6D). In contrast to HER2, staining for pHER3 was weak but the lowest IRS scoring was seen in the combination arm (Supplementary Figure 3B). Consistent with the cell line data, neratinib and trastuzumab inhibited pAkt to a greater extent than trastuzumab monotherapy but not neratinib monotherapy (Figure 6E and Supplementary Figure 3C). Neratinib treatment showed little effect on ERK phosphorylation whereas trastuzumab alone and the combination treatment decreased pERK staining in the xenograft tumors (Figure 6F and Supplementary Figure 3C). However, the differences in pHER3, pAkt and pERK IHC staining were not statistically significant.

## DISCUSSION

Our results showed that the combination of trastuzumab and neratinib treatment was significantly more potent at reducing cell viability than trastuzumab alone in both sensitive and acquired resistant HER2 over-expressing SKBR3 and BT474 breast cancer cells. In the trastuzumab-naïve SKBR3 and BT474 cells, acute neratinib treatment inhibited phosphorylation of EGFR, HER2, HER3 and HER4 as well as downstream pathways ERK and Akt, reflecting its immediate inhibitory effect on the tyrosine kinase activity of all the HER receptors. In contrast, trastuzumab did not decrease phosphorylation of EGFR, HER2, HER4 and ERK, reflecting the different mechanisms of action of the drugs. The xenograft experiment also showed that the combination treatment lead to the greatest decrease in pHER2 with decreased activation of pAkt and pERK, correlating with increased efficacy compared to the single agents, in xenograft models. Although trastuzumab has been previously shown to downregulate HER2 [12, 19, 31], this effect was not seen with either trastuzumab, neratinib or the combination treatment in our xenograft study. This may be because there was a significant heterogeneity in HER2 staining between xenograft tumor areas and the dose of trastuzumab used in this study was lower than in previously reported xenograft experiments [31], which may affect the amount of HER2 downregulation and assessment [32].

Clinically, the withdrawal of trastuzumab treatment in patients who are no longer responding is controversial [33], partially due to the cost of continuing trastuzumab treatment [34]. Our data revealed that the withdrawal of trastuzumab from the trastuzumab-resistant cell lines resulted in a significantly increased cell count compared to continuation of trastuzumab treatment. Furthermore, the combination of trastuzumab and neratinib was significantly more effective than neratinib alone even in the presence of trastuzumab resistance. This is supported by recent data which showed that lapatinib in combination with trastuzumab significantly improved overall survival and progression-free survival compared to lapatinib alone despite disease progression on prior trastuzumab-based therapy [35, 36].

Neratinib has shown promising activity in several clinical trials, particularly in HER2 positive breast cancer patients [29, 37]. Neratinib and trastuzumab in combination with paclitaxel chemotherapy showed clinical benefit in patients who had been heavily pre-treated with anti-HER2 agents and chemotherapy, with a mean of 4 prior regimens [38]. A phase II multi-centre randomized study of neratinib in combination with weekly paclitaxel with or without trastuzumab followed by doxorubicin and cyclophosphamide (AC) as neoadjuvant therapy for women with HER2-positive locally advanced breast cancer (NSABP FB-7) is currently ongoing. As well as neratinib, other irreversible TKIs including dacomitinib (PF-00299804) and afatinib (BIBW2992) are also being tested in clinical trials. Afatinib monotherapy was shown to have promising clinical activity in extensively pretreated HER2-positive breast cancer patients who had progressed following trastuzumab treatment [39].

It has been previously shown that EGFR and HER2 inhibitors failed to suppress HER3 phosphorylation due to ligand-dependent activation of HER3 via ADAM17 through an Akt feedback loop [19, 40, 41]. This could be an important drug resistance mechanism for EGFR and HER2 inhibitors. We showed that although neratinib effectively inhibited phosphorylation of all HER receptors for up to 24 hours, reactivation of HER3 and Akt occurred within 3 days. However, the combination of trastuzumab and neratinib significantly delayed reactivation of HER3 and Akt compared to neratinib alone. In contrast a recent study showed that dual inhibition with trastuzumab and lapatinib or trastuzumab and pertuzumab does not completely eliminate HER3 function [42]. It would be interesting to determine if the reactivation of HER3 observed with neratinib is also induced by ADAM17 mediated heregulin release [19]. This possibility might be investigated using an ADAM17 inhibitor or a monoclonal antibody to HER3.

Although neratinib is a panHER inhibitor, we showed that it is selectively active in HER2 amplified compared to non-amplified breast cancer cell lines. However, some of the HER2 amplified cells listed in Supplementary Table1, including MDA-MB-361 and MDA-MB-453, have been classified as IHC 2+ or HER2 negative [43-46], as they express slightly lower levels of HER2 protein, at an expression level that is nearer to the original FDA definition of HER2 positivity [47]. Since HER2 testing results may be variable according to the methods or assays of testing [48], it is not surprising that some of these cells have been classified differently. Nevertheless, some of the moderate HER2 expressing breast cancer cells including MDA-MB-453 and MDA-MB-361 show greater sensitivity to neratinib than trastuzumab. In NSABP B-41, lapatinib in combination with chemotherapy induced a greater pathological complete response than that of trastuzumab in combination with chemotherapy in breast cancer patients with IHC 2+ although the number of patients in each arm was small [49]. Thus it may be important to assess the effect of neratinib in patients whose tumors express lower levels of HER2 protein (IHC 2+).

Despite the frequent overexpression of EGFR in triple negative breast tumors and cell lines, they were less sensitive to neratinib than HER2 amplified cells. The luminal cell line, MDA-MB-175, which showed significant sensitivity to neratinib has previously been shown to be dependent on heregulin-mediated activation of HER2/HER3 signaling and are also more sensitive to pertuzumab treatment than trastuzumab [50]. In addition, neratinib but not lapatinib, has been shown to be active against HER2 somatic mutations present in about 1-3% of HER2 negative breast cancer patients [51]. This suggests that there may be both overlapping and non-overlapping mechanisms of resistance to different HER2 inhibitors. There is a need to further understand the biomarkers that can predict response to neratinib in different breast cancer subtypes.

The levels of total HER2 protein and phosphorylated HER2 correlated with response to neratinib in the panel of HER2 overexpressing cell lines, similar to lapatinib [30]. HER2 protein and mRNA levels have also been shown to predict response to dacomitinib [52]. The reduction in pAkt and pERK in response to neratinib treatment was also correlated with sensitivity to neratinib. Other potential biomarkers which may be predictive of response to neratinib, and other HER2 targeted therapies, include the dimerization states of the HER receptors [53], and levels of protein tyrosine phosphatases, such as PTPN9 which has been shown to regulate EGFR and HER2 activation [54, 55].

Like lapatinib [30], neratinib can overcome trastuzumab resistance in cell line models of acquired trastuzumab resistance and can enhance response to trastuzumab in trastuzumab sensitive cell lines. However, neratinib also shows activity in some cell lines which are innately resistant to lapatinib which suggests that neratinib may have clinical benefit in patients who do not respond to trastuzumab and/or lapatinib. The challenge will be to identify predictive markers to select those patients with HER2 positive breast cancer who may benefit from neratinib treatment but not from trastuzumab or lapatinib. The results of this study provide a rational for clinical trials of neratinib in combination with trastuzumab and of neratinib alone (with chemotherapy) in patients whose disease has progressed on trastuzumab and/or lapatinib treatment.

## MATERIALS AND METHODS

### Cell lines and generation of trastuzumab resistant cell lines

SKBR3 and BT474 were obtained from CRUK London Research Institute Cell Services, and were cultured in RPMI and DMEM respectively; both were supplemented with 10% FBS, penicillin-streptomycin. Trastuzumab-resistant BT474 and SKBR3 cell lines were generated as previously described [19]. In addition to SKBR3 and BT474, the following panel of breast cancer cell lines was used in this study: EFM192A, HCC1419, MDA-MB-361, MDA-MB-453, SUM190, SUM225, UACC732, UACC812, UACC893, CAMA1, EFM19, KPL1, MCF7, MDA-MB-134, MDA-MB-175, MDA-MB-415, T47D, ZR751, HCC1569, HCC1954, BT20, BT549, CAL51, HCC38, HCC70, HCC1143, HCC1187, HCC1395, HCC1806, HCC1937, JIMT1, MDA-MB157, MDA-MB-231, MDA-MB-468, MDA-MB-435, MDA-MB-436. All cell lines used were obtained from the American Type Culture Collection (Rockville, MD, USA) unless otherwise stated. EFM192A, KPL1, EFM19 and CAL51 were supplied by the German Tissue Repository DSMZ (Braunschweig, Germany). SUM190 and SUM225 were obtained from the University of Michigan (Ann Arbor, MI, USA). MDA-MB-134, MDA-MB-415, MDA-MB-436, MDA-MB-157, UACC893 and UACC812 were cultured in L15 medium supplemented with 10 % heat-inactivated fetal bovine serum (FBS), 2 mM glutamine and 1 % penicillin G-streptomycin-fungizone solution (PSF) (Irvine Scientific, Santa Ana, CA, USA). DMEM (Cellgro, Manassas, VA, USA) supplemented with 10 % heat-inactivated FBS and PSF (Irvine Scientific, Santa Ana, CA, USA) was used to culture CAL51 and KPL1. SUM190 and SUM225 were maintained in HAM's F12 supplemented with 5 % heat inactivated FBS, PSF, 5 mg/ml insulin and 1 mg/ml hydrocortisone. All other cell lines were maintained in RPMI 1640 (Cellgro) supplemented with 10 % FBS (heat inactivated), 2 mM glutamine and 1 % PSF (Irvine Scientific, Santa Ana, CA, USA). All cell lines were grown in a humidified incubator at 37 °C with 5 % CO_2_ apart from MDA-MB-134, MDA-MB-157, MDA-MB-175, MDA-MB-361, MDA-MB-415, MDA-MB-436, UACC812 and UACC893 which were grown at 37 °C in the absence of CO_2_.

Stock solutions of lapatinib (10 mM) (Sequoia Research Products) and neratinib (10 mM) (supplied by Pfizer) were prepared in dimethyl sulfoxide (DMSO). Trastuzumab was purchased from St Vincent's University Hospital and Oxford University Hospitals NHS Trust.

### Proliferation assays

#### Proliferation was assessed using cell counting and acid phosphatase assays

For cell counting experiments, cells were seeded in duplicate at 2 – 3 × 10^4^ cells per well, depending on the cell line, in 24 well plates. The cells were treated for 5 days unless otherwise stated. On the concluding day of the experiments, the cells were washed with phosphate buffered saline (PBS) before trypsinization and counted using ISOTON solution on the Coulter Z2 particle counter (Beckham Coulter, Inc). Growth inhibition relative to control treated cells was determined. The log of drug concentration was then plotted against the log growth inhibition and IC_50_ values were calculated using linear regression analysis as previously described [30].

For the acid phosphatase assay, 5 × 10^4^ cells/well for MDA-MB-361 cells, and 3 × 10^4^ cells/well for the other cell lines were seeded in triplicate 96-well plates. Following overnight incubation at 37°C, drugs were added at the appropriate concentrations and incubated for 5 days at 37°C. Cells were washed once with PBS and acid phosphatase substrate (7.25 mM p-nitrophenyl-phosphate (Sigma) in sodium acetate buffer) was added to each well and incubated at 37 ºC for 1 hour. The reaction was stopped by adding 50 μl 1 M NaOH and absorbance was read at 405 nm with 620 nm as the reference wavelength.

### Xenograft experiment

The flank of BALC/nu/nu mice was injected with 10^7^ BT474 cells in Matrigel and left to grow (under the project license PPL 30/2771). Mice were provided with 5 μg/ml oestradiol in the drinking water for the duration of the experiment. When tumors reached an average size of 125 mm^3^, the mice were divided into 4 groups, keeping average tumor size similar between groups. The control group of 4 mice was treated with vehicle (0.5% methocellulose - 0.4% polysorbate-80 (Tween 80)) by gavage daily and sterile PBS intraperitoneally (IP) twice weekly. The treatment groups consisted of 5 mice, treated with: (i) vehicle containing neratinib 10 mg/kg by gavage daily and IP PBS twice a week; (ii) trastuzumab 10 mg/kg (twice weekly IP) and vehicle by gavage daily; (iii) trastuzumab 10 mg/kg by IP twice a week with neratinib (10 mg/kg) by gavage daily. The treatment continued for 17 days (5 trastuzumab treatments) and the mice were sacrificed 3 hours after the last neratinib treatment. Tumours were weighed and collected in 4% formalin to fix over night at 4 °C. The samples were then embedded in paraffin for immunohistochemical analysis.

### Western Blotting

#### Neratinib dose escalation studies in naïve and acquired resistant SKBR3 and BT474 cells

SKBR3 and BT474 cells were seeded and left to adhere overnight. Cells were treated as indicated before they were placed on ice and washed with PBS. Lysis buffer (10 mM EDTA, 20 mM Tris pH 7.5, 150 mM NaCl, 10 mM Na_2_P_2_O_7_ and 100 mM NaF with 1% Triton X and 1:100 protease inhibitor cocktail) was added to the cells and the cells scraped off the plates. Samples were centrifuged at 4°C to remove the insoluble cell pellets. Bradford Protein Assay was then performed to determine protein concentration. Equal amounts of protein sample were prepared in 4X SDS with 10% beta-mercaptoethanol, boiled for 10 minutes at 95°C. Samples were then loaded into a NuPage 4-12% gel and ran at 100V for 15 minutes then 130V until completion. The proteins were semi-dry transferred to a PVDF membrane for two hours at 12V. The membrane was blocked in 3% BSA in PBS-Tween (0.2%) for a minimum of one hour. The membrane was then incubated with primary antibody in 3% BSA in PBS-Tween overnight at 4°C. All antibodies were purchased from Cell Signalling Technologies. The membrane was then washed four times with 1% milk in PBS-Tween (0.2%) at room temperature and incubated with secondary antibody in 5% milk PBS-Tween (0.2%) for one hour before washing four times in 1% milk PBS-Tween. Bands were then visualized and the blot developed using an enhanced chemiluminescent system (ECL, GE Heathcare). Bands were quantified using ImageJ software.

#### Pharmacodynamic biomarkers of neratinib response in a panel of cell lines

After neratinib treatment for 24h, cells were washed with PBS and lysed in RIPA buffer (Sigma) containing protease inhibitors, 1 mM phenylmethylsulfonyl fluoride (PMSF) and 1 mM sodium orthovanadate. After 20 min incubation on ice, lysate was passed through a 21-gauge needle and centrifuged at 10,000 g for 5 min at 4ºC. Protein quantification was carried out using the bicinchoninic acid assay (Pierce).

Forty μg of protein were solubilized in sample buffer (250 mM Tris–HCl; 10% sodium dodecyl sulphate (SDS); 5% beta-mercaptoethanol; 30% glycerol; 0.02% bromophenol blue), heated to 95 ºC for 5 min and separated using 7.5% polyacrylamide gels (Lonza). Proteins were transferred to Hybond enhanced chemiluminescence nitrocellulose membrane (Amersham, Biosciences, Buckinghamshire, UK). The membrane was blocked with 5% milk in PBS–0.1% Tween at room temperature for 1h. After overnight incubation at 4ºC with primary antibody (anti-HER2, Calbiochem, all other antibodies from Cell Signaling Technology) in 5% blocking solution milk in PBS–0.1% Tween, three washes with PBS–0.1% Tween were carried out, followed by incubation at room temperature with secondary antibody in 5% blocking solution milk in PBS–0.1% Tween for 1 hour. Following three washes with PBS–0.1% Tween and one PBS wash, protein bands were detected using Luminol (Santa Cruz Biotechnology Inc., Santa Cruz, CA).

### Immunohistochemisty and immunoreactive (IRS) scoring

Formalin-fixed BT474 xenograft samples were embedded in paraffin and 4 μm thick sections were cut for immunohistochemical staining. The sections were dewaxed and rehydrated prior to antigen retrieval using Tris/EDTA at pH9 for HER2 and phosho-HER2, or citrate buffer at pH6 for the other antibodies. Sections were blocked using horse serum (Vector laboratories) overnight at 4ºC. The following day, sections were incubated with primary antibodies for 2 hours at room temperature, followed by 3 washes with PBS. The sections were then incubated with a horseradish peroxidise-coupled anti-rabbit secondary antibody (Vector laboratories) for 30 minutes at room temperature. Visualization of staining was performed using a diaminobenzidine (DAB) peroxidase substrate (Vector Laboratories). Counterstaining was performed using Haemocytoxin prior to the mounting using aqueous-based mounting media (Aquatex). Levels of staining were scored blindly. The scoring criteria is composite score based on staining intensity (SI) and percentage of positive cells (PP) using the formula IRS = SI × PP. The staining intensity (SI) was determined as 0 = negative; 1 = weak; 2 = moderate; and 3 = strong and the percentage of positive cells (PP) was defined as 0, <1%; 1, 1%–10%; 2, 11%–50%; 3, 51%–80%; and 4, >80% positive cells.

### Statistical analysis

Mann-Whitney *U* test was used to investigate the association between response to neratinib and breast cancer cell line subtype. The differences in the means between different conditions or groups (n>2) were analysed by Anova with Bonferroni's multiple comparison test and p < 0.05 was taken to be statistically significant. Relationships between neratinib response and HER2, pHER2, p95, EGFR, HER3, Akt, ERK, PTEN, PI3K and ER (from O'Brien *et al* [30]) were examined using the Spearman's Rank correlation and Mann-Whitney *U* test on StatView for Windows (version 5.0.1) (SAS Institute, Inc).

## Supplementary Figure and Tables




