# Supplementary material for: Neratinib overcomes trastuzumab resistance in HER2 amplified breast cancer
Source: Oncotarget. 2013 Jul 25;4(10):1592–605. doi: 10.18632/oncotarget.1148 (PMC3858548; doi:10.18632/oncotarget.1148)
Supplement: Supplementary file 1 [file oncotarget-04-1592-s001.pdf]

Neratinib overcomes trastuzumab resistance in HER2 amplified breast cancer - Canonici et al

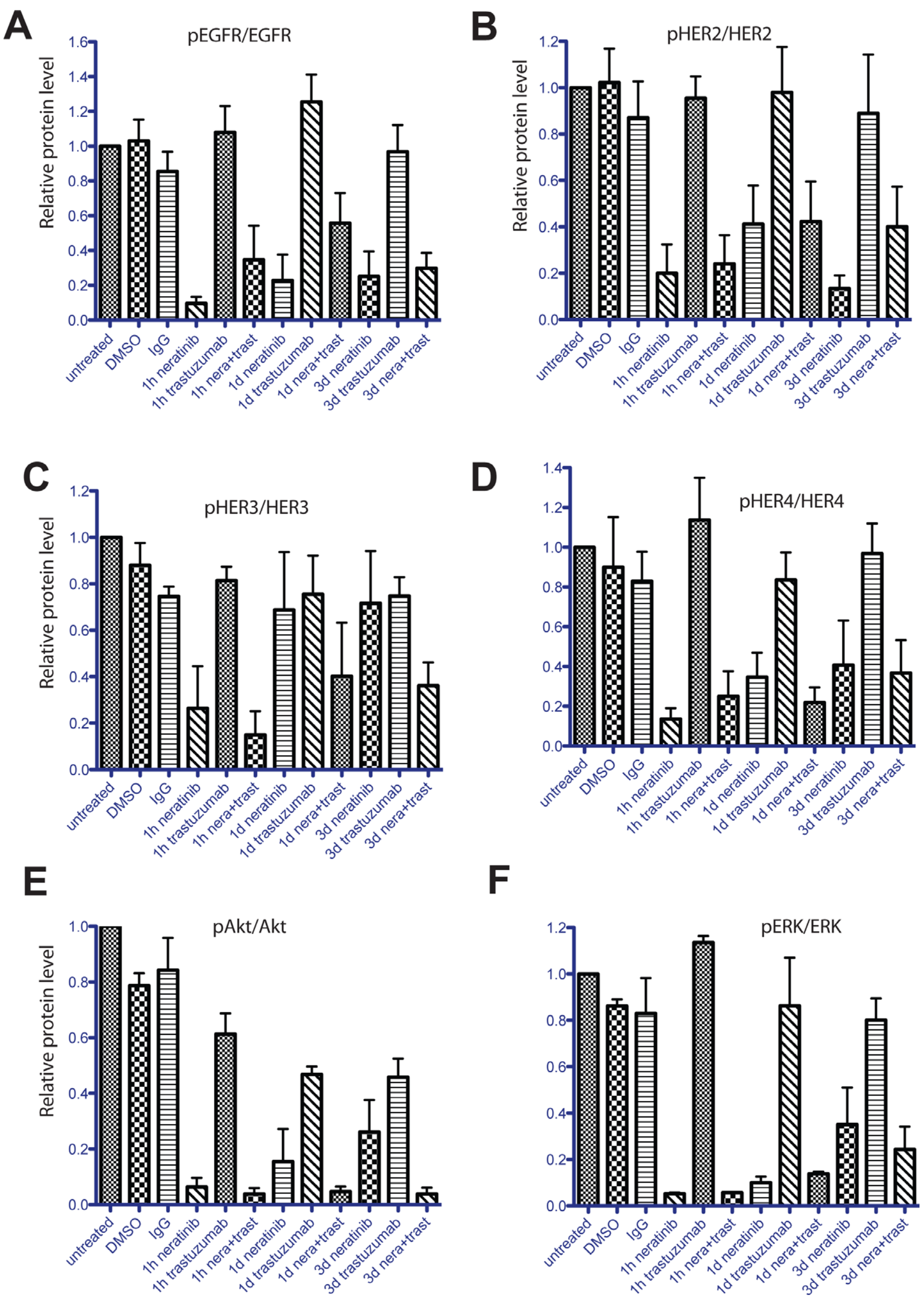

Supplementary Figure 1: Combination of neratinib and trastuzumab has an additive effect and prevents re-activation of pHER3 and pAkt. BT474 and SKBR3 cells were treated with 2 nM neratinb, 40 µg/ml trastuzumab or their combination for the indicated times before the cells were lysed and analysed by western blot. Western blot lanes of three independent experiments from Figure 2b were quantified using ImageJ software. Ratios between total levels of protein and phosphorylated proteins were calculated.

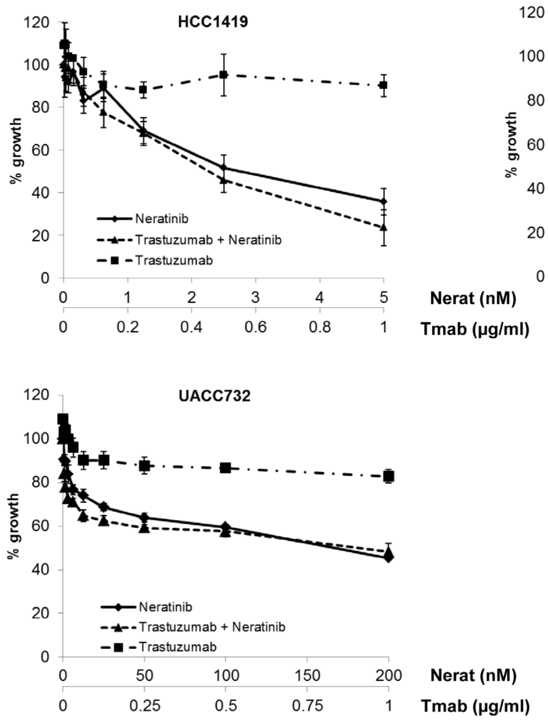

Supplementary Figure 2: Combined neratinib and trastuzumab in cell line models of innate trastuzumab resistance. Sensitivity to trastuzumab and neratinib in three innately trastuzumab resistant HER2 positive breast cancer cell lines. Cells were treated with neratinib alone, trastuzumab alone or the combination at a fixed ratio.

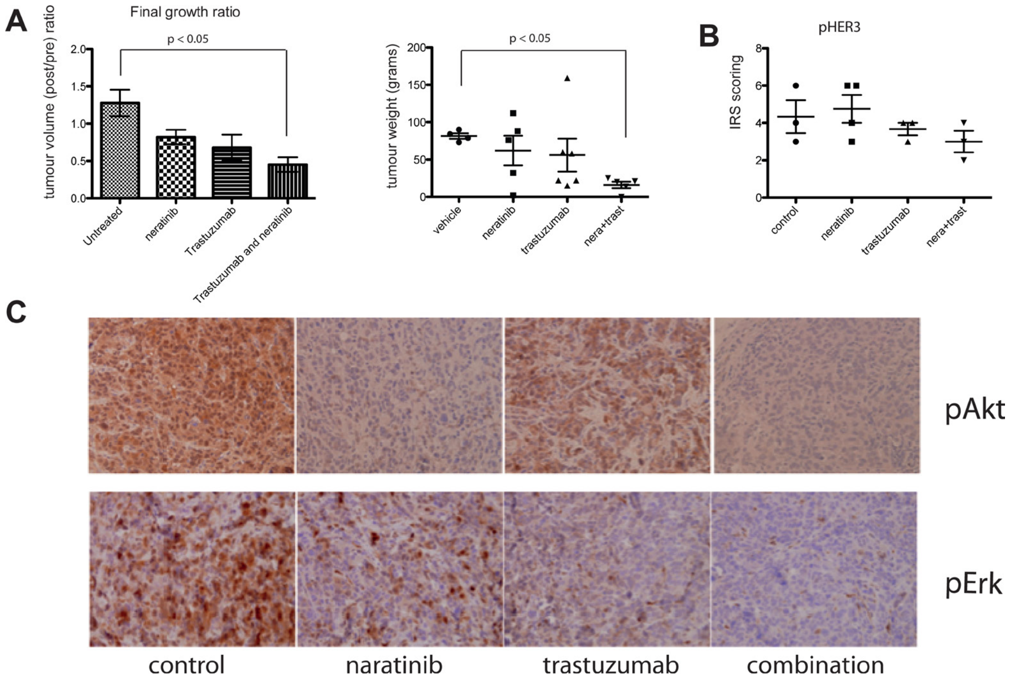

Supplementary Figure 3: The combination of neratinib with trastuzumab is most effective in reducing tumor growth and downstream pathways. A) Right, mice bearing BT474 xenograft tumors were treated with either indicated conditions and at the end of the experiment, tumor samples were collected and weighed. Left, the ratios of the post-treatment over pre-treatment tumor volume of the different groups are illustrated in the figure. The differences in the means between the groups were analyzed by Anova with Bonferroni's multiple comparison test and statistically significant changes were indicated in the figures. B-C) Sections were cut from paraffin-embedded xenograft samples and stained for phopshorylated HER3 (pHER3), phosphorylated Akt (pAkt) and phosphorylated ERK (pERK). For pHER3 staining, one control sample was excluded due to no staining and one neratinib treated sample was excluded due to artefact. Representative photos of pAkt and pERK from each condition are shown in C.
