# Supplementary material for: Neratinib overcomes trastuzumab resistance in HER2 amplified breast cancer
Source: Oncotarget. 2013 Jul 25;4(10):1592–605. doi: 10.18632/oncotarget.1148 (PMC3858548; doi:10.18632/oncotarget.1148)
Supplement: Supplementary file 2 [file oncotarget-04-1592-s002.pdf]

Supplementary Table 1. Classification of breast cancer cell line subtypes and response to neratinib (IC_{50}).

Cell Line	IC_{50} value (µM)	IC_{50} SE	Breast Cancer Subtype	ER Status
**BT-474**	<0.005	n/a	HER2+	Positive
**EFM-192A**	<0.005	n/a	HER2+	Positive
**HCC1569**	<0.005	n/a	HER2+	Negative
**HCC1954**	<0.005	n/a	HER2+	Negative
**MDA-MB-175**	<0.005	n/a	Luminal	Positive
**MDA-MB-361**	<0.005	n/a	HER2+	Positive
**SK-BR-3**	<0.005	n/a	HER2+	Negative
**UACC-812**	<0.005	n/a	HER2+	Positive
**UACC-893**	<0.005	n/a	HER2+	Positive
**SUM-225**	0.01	0.00	HER2+	Negative
**SUM-190**	0.01	0.00	HER2+	Positive
**ZR-75-1**	0.03	0.02	Luminal	Positive
**HCC70**	0.03	0.20	Triple Negative	Negative
**BT-20**	0.07	0.04	Triple Negative	Negative
**MDA-MB-453**	0.09	0.00	HER2+	Negative
**HCC1187**	0.10	0.07	Triple Negative	Negative
**EFM-19**	0.11	0.07	Luminal	Positive
**T-47D**	0.16	0.04	Luminal	Positive
**MDA-MB-134**	0.17	0.00	Luminal	Positive
**HCC38**	0.25	0.12	Triple Negative	Negative
**MDA-MB-435**	0.33	0.06	Triple Negative	Negative
**MDA-MB-468**	0.33	0.80	Triple Negative	Negative
**CAMA-1**	0.37	0.52	Luminal	Positive
**MDA-MB-436**	0.41	0.00	Triple Negative	Negative
**MCF-7**	0.41	0.03	Luminal	Positive
**MDA-MB-415**	0.42	0.00	Luminal	Positive
**HCC1806**	0.44	0.13	Triple Negative	Negative
**HCC1395**	0.49	0.52	Triple Negative	Negative
**HCC1937**	0.50	0.26	Triple Negative	Negative
**HCC1143**	0.54	0.19	Triple Negative	Negative
**UACC-732**	0.65	0.37	HER2+	Positive
**MDA-MB-231**	1.00	0.17	Triple Negative	Negative
**MDA-MB-157**	1.12	0.00	Triple Negative	Negative
**BT-549**	1.14	0.53	Triple Negative	Negative
**KPL-1**	1.89	0.16	Luminal	Positive
**CAL-51**	1.89	2.06	Triple Negative	Negative

Abbreviation: n/a – not applicable

Supplementary Table 2. **Relationship between neratinib sensitivity (IC_{50} values) and potential biomarkers in a panel of 11 HER2 amplified breast cancer cell lines.** Correlations were performed using the Spearman's Rank or Mann Whitney *U* test.

Marker	p value	r
**EGFR**	0.720	0.115
**pEGFR**	0.071	-0.600
**HER2**	0.038	-0.665
**p-HER2**	0.042	-0.643
**p95-HER2**	0.098	-0.523
**HER3**	0.796	-0.820
**pHER3**	0.433	-0.248
**IGF-IR**	0.239	0.373
**p-IGF-IR**	0.478	-0.236
**AKT**	0.863	-0.055
**pAKT**	0.546	-0.191
**p27**	0.966	0.014
**PI3K status**	0.340	NA*
**PTEN status (low/high)**	0.200	NA
**ER status**	0.140	NA

*NA – not applicable
